# Acute hypersensitivity reactions to paclitaxel polymeric micelles during malignant tumor treatment: a retrospective real-world study

**DOI:** 10.3389/fonc.2026.1908304

**Published:** 2026-08-18

**Authors:** Xing Xia, Dongliang Han, Lin Wang, Haifei Yang, Ansu Li, Yan Chen, Wu Sun, Wei Ren

**Affiliations:** 1 Department of Pharmacy, Nanjing Drum Tower Hospital, Affiliated Hospital of Medical School, Nanjing University, Nanjing, China; 2 Department of Oncology, Nanjing Drum Tower Hospital Clinical College of Nanjing University of Chinese Medicine, Nanjing, China; 3 Department of Oncology, Nanjing Drum Tower Hospital, Affiliated Hospital of Medical School, Nanjing University, Nanjing, China; 4 Department of Clinical Laboratory, Nanjing Drum Tower Hospital, Affiliated Hospital of Medical School, Nanjing University, Nanjing, China

**Keywords:** acute hypersensitivity reaction, clinical characteristics, malignant tumor, paclitaxel polymeric micelle, real-world study, retrospective study

## Abstract

**Background and aims:**

Paclitaxel polymeric micelles (pm-Pac) are widely used for solid tumors, but real-world data on acute hypersensitivity reactions (HSRs) are limited. This exploratory study aimed to characterize the clinical features, risk factors, and dynamic laboratory changes in pm-Pac-associated acute HSRs.

**Methods:**

This retrospective study included 220 patients treated with pm-Pac from March 2023 to April 2026. Clinical data were extracted from electronic medical records. Analyses included chi-square tests, linear-by-linear association, logistic regression, and linear mixed-effects models.

**Results:**

The overall HSR incidence was 6.8% (15/220). Most HSRs were mild-to-moderate (grade 1: 40.0%; grade 2: 33.3%) and 26.7% were grade 3. Reactions occurred predominantly within the first 60 minutes (60.0%); 88.9% of moderate-to-severe reactions (grade ≥2) occurred within this window. Fourteen events (93.3%) occurred during the second cycle, and one (6.7%) during the third cycle, with no recurrence in six patients who continued treatment. Rash and pruritus were most common manifestations, followed by cardiovascular symptoms. Conventional risk factors showed no significant associations, and routine laboratory parameters were similar between groups. Baseline autoantibody positivity was 66.7% in HSR compared with 41.2% in non-HSR patients (*P=*0.055). HSR severity grade was correlated with treatment outcome (*P=*0.001; improvement rates were 0% for grade 1, 100% for grade 2, and 66.7% for grade 3). Among evaluable patients receiving a glucocorticoid-antihistamine-calcium gluconate combination, 85.7% improved.

**Conclusions:**

Pm-Pac-induced acute HSRs were predominantly mild-to-moderate cutaneous reactions, occurring within the first 60 minutes and during the second cycle; grade ≥2 reactions were more frequent in the early time window. No recurrent reactions were observed in subsequent cycles. Routine laboratory monitoring did not differentiate between patients with and without HSRs; symptom surveillance is essential. Baseline autoantibody positivity showed a non-significant trend that requires validation. Given the small sample (n=15), all findings are exploratory and require confirmation in larger cohorts.

## 1 Introduction

Paclitaxel polymeric micelles (pm-Pac), a novel paclitaxel formulation, encapsulate paclitaxel within polymeric micelles, significantly improving the drug’s water solubility while reducing the risk of non-immune-mediated hypersensitivity reactions (HSRs) associated with the solubilizer (Cremophor EL) used in conventional paclitaxel injections (1, 2). Pm-Pac has a stable core-shell structure measuring 18–20 nm that facilitates tumor targeting via the enhanced permeability and retention (EPR) effect, thereby improving efficacy and reducing toxicity. Importantly, its maximum tolerated dose (390 mg/m²) and dose-limiting toxicity threshold (435 mg/m²) are the highest among all taxanes, enabling safe dose escalation (1, 2). In phase III clinical trials of first-line treatment for advanced non-small cell lung cancer (NSCLC), pm-Pac demonstrated a higher objective response rate and superior progression-free survival compared with other taxane formulations. As a result, it has been widely adopted not only for NSCLC but also for other solid malignancies, including breast and ovarian cancers (3–5).

Nevertheless, HSRs remain a serious and often under-recognized adverse event associated with paclitaxel-based chemotherapy (6). The reported incidence of HSRs with conventional paclitaxel ranges from 1.2% to 42% (7–9). This variability may be attributed to differences in study populations, premedication regimens, and HSR definitions. Most HSRs occur during the first or second infusion, with clinical manifestations ranging from mild rash and pruritus to nausea, vomiting, and even anaphylactic shock (9, 10). Standard premedication reduces the risk, but reactions still occur, and cumulative exposure may increase the risk across successive cycles (11). In recent years, retrospective studies of other antineoplastic agents, such as oxaliplatin and carboplatin, have provided detailed characterizations of HSR clinical features and have developed skin-testing and desensitization protocols to guide rechallenge (12–14). However, real-world data on acute HSRs to pm-Pac remain scarce, particularly regarding multidimensional analyses incorporating dynamic laboratory changes.

Although pm-Pac eliminates the Cremophor EL-related risk of severe hypersensitivity and does not require routine premedication according to the package insert, sporadic acute HSRs to pm-Pac have been reported (15, 16), and we have observed previously unreported severe cases in clinical practice. When managing pm-Pac -associated HSRs, physicians often lack local data on incidence, risk factors, and laboratory reference ranges, hindering the development of precise prevention and management strategies.

To address this knowledge gap, we conducted a retrospective analysis of clinical data from 220 patients with malignant tumors treated with pm-Pac. We systematically described the clinical characteristics of acute HSRs (including time to onset, symptoms, and severity grading), explored potential risk factors (with particular emphasis on the cumulative effect of treatment cycles), analyzed dynamic changes in laboratory parameters between the allergic and non-allergic groups across multiple measurements, and evaluated HSR management and outcomes. We aimed to provide preliminary real-world evidence that may inform future risk stratification and clinical management of pm-Pac-associated HSRs.

## 2 Methods

### 2.1 Study design and ethics

This retrospective, real-world cohort study aimed to characterize the clinical features of acute HSRs in patients receiving pm-Pac therapy for malignant tumors and to identify associated risk factors. The study protocol was approved by the Ethics Committee of Nanjing Drum Tower Hospital (Approval No.: 2025124501) and was conducted in strict accordance with the ethical principles of the Declaration of Helsinki. All patient information was anonymized to ensure privacy and data confidentiality. Given the retrospective nature and complete anonymization of the data, the Ethics Committee waived the requirement for informed consent.

### 2.2 Data sources and study subjects

Clinical data were collected from consecutive patients with malignant tumors who received pm-Pac therapy at Nanjing Drum Tower Hospital between March 2023 and April 2026. Data were extracted from the hospital’s electronic medical record system. Baseline characteristics, treatment information, laboratory test results, and adverse event records were entered into a standardized data collection platform by trained researchers.

#### 2.2.1 Inclusion criteria

Patients were included if they met the following criteria: (1) patients with confirmed pathological or cytological diagnosis of a malignant tumor; (2) treatment with pm-Pac as monotherapy or as part of combination chemotherapy; (3) for patients with an acute HSR, clinical symptoms meeting the diagnostic criteria for HSRs occurring from the start of pm-Pac infusion to 6 hours after; such symptoms included but not limited to mucocutaneous manifestations (rash, pruritus, flushing), respiratory symptoms (dyspnea, wheezing, laryngeal edema), cardiovascular signs (hypotension, tachycardia), and gastrointestinal symptoms (nausea, vomiting, abdominal pain); and (4) patients with complete clinical data, including baseline information (age, sex, medical history), treatment-related data (administration cycles and premedication regimens), HSR-related records (time of onset, symptoms), and follow-up information.

#### 2.2.2 Exclusion criteria

Patients were excluded if they met any of the following criteria: (1) HSRs clearly attributable to concomitant medications, excipients, or factors other than pm-Pac; (2) HSRs occurring more than 6 h after pm-Pac administration; and (3) incomplete clinical data (missing critical information, such as time of onset, symptoms, and management measures). The attribution of HSRs to pm-Pac versus other agents (e.g., cisplatin) was determined by the attending oncologists based on temporal association with the pm-Pac infusion, symptom pattern consistent with taxane-induced HSRs, and exclusion of other identifiable causes. In addition, the infusion sequence was standardized, with pm-Pac administered first over 3–4 hours, followed by cisplatin. All HSRs occurred during the pm-Pac infusion phase.

### 2.3 Data collection and variable definitions

#### 2.3.1 Clinical data collection

Data were collected retrospectively using a standardized case report form and included the following: (1) general demographic characteristics: age, sex, body mass index (BMI), tumor type, and stage; (2) medical history: history of allergies (drug allergies, food allergies, and other allergic diseases, such as allergic rhinitis, asthma) and other comorbid diseases (pulmonary disease, cardiovascular disease); (3) treatment-related information: administration regimen (including the number of treatment cycles) and premedication regimen (use of corticosteroids or antihistamines); (4) HSR information: (i) time interval (in minutes) from the start of pm-Pac administration to the first appearance of HSR symptoms. (ii) clinical manifestations, including mucocutaneous (facial flushing, rash, urticaria, pruritus), respiratory (chest tightness, dyspnea, wheezing, laryngeal edema, cyanosis), cardiovascular (palpitations, hypotension, tachycardia, circulatory failure), and gastrointestinal (nausea, vomiting, abdominal pain, diarrhea) symptoms. (iii) severity grading according to *A Clinical Practice Guideline for the Emergency Management of Anaphylaxis (2020)* (17); (5) laboratory parameters: results of routine blood tests (including white blood cell and eosinophil counts), liver function tests (alanine aminotransferase and aspartate aminotransferase), renal function tests (serum creatinine), and inflammatory markers (C-reactive protein) were recorded at the time of the HSR and following treatment. All patients received pm-Pac 230 mg/m² in combination with cisplatin 70 mg/m² in cycle 1. In subsequent cycles, the pm-Pac dose was escalated to 300 mg/m² if cycle 1 was well tolerated (defined as neutrophil nadir >1.0 × 10^9^/L, platelet nadir >80 × 10^9^/L, and no grade 2–4 non-hematologic toxicity), in accordance with the Chinese guidelines for esophageal carcinoma.

After the initial HSR, follow-up data were recorded for each subsequent cycle, including symptom assessments during infusion and within 6 hours post-infusion. The observation period for recurrence extended from the initial HSR to the last documented follow-up; the median follow-up duration was 4.0 months (range: 2.5–8.5 months).

#### 2.3.2 Outcome measures

The primary outcome was the characterization of clinical features of acute HSRs, including (1) time of onset distribution (categorized in minutes, e.g., ≤60 min, >60 min); (2) symptom presentation; (3) severity grade distribution; (4) response rate to treatment; and (5) time to resolution.

The secondary outcome was the identification of risk factors associated for acute HSRs to pm-Pac, including age, sex, BMI, history of allergies, and other comorbid diseases.

Safety outcomes included adverse events that occurred during HSR management and were recorded and analyzed.

### 2.4 Statistical analysis

All analyses were performed using SPSS 26.0 (IBM Corp., Armonk, NY, USA). Normality was assessed using the Shapiro-Wilk test. Continuous variables are presented as mean ± standard deviation or median (interquartile range), and categorical variables as n (%). For between-group comparisons, t-tests, Wilcoxon rank-sum tests, chi-square tests, or Fisher’s exact test were used as appropriate. The linear-by-linear association chi-square test was used to assess trends between ordered variables and HSR occurrence. Linear mixed-effects models were used to analyze repeated laboratory measurements, with group, time, and their interaction as fixed effects and patient ID as a random effect. Univariate logistic regression was used to screen for potential risk factors. Two-sided tests were applied, and *P <* 0.05 was considered statistically significant. Given the limited number of HSR events (n = 15), this was an exploratory analysis without correction for multiple comparisons; results with *P values* between 0.05 and 0.10 were considered borderline.

## 3 Results

### 3.1 Baseline characteristics and incidence of hypersensitivity reactions

A total of 220 patients with malignant tumors who received pm-Pac were included in the study. Among them, 15 patients (6.8%) experienced acute HSRs, whereas 205 patients (93.2%) did not. The baseline characteristics are presented in **Table 1**. The median age of the entire cohort was 69 years (IQR 62–73 years), and patients aged ≥60 years accounted for 82.3%. Males comprised 84.5% of the study population. No statistically significant differences were observed between the HSR and non-HSR groups in age, cancer stage, sex, BMI, history of allergies, prior medical history, autoantibodies, number of drug administrations, or premedication (*P* > 0.05). Among the 17 patients with a history of allergies, only 1 (5.9%) developed an HSR, which was not significantly different from the incidence in patients without such a history (6.9%, 14/203, *P* = 1.000).

**Table 1:** Patient baseline characteristics of the study population.

Variables	Total population (N 220)	HSR group (N 15)	Non-HSR group (N 205)	*P-value*
Demographics				
Age, years ^1^				
Median (IQR)	69 (62–73)	66 (62–72)	69 (62–73)	^1^0.417
< 60 years, n (%)	39 (17.7)	1 (6.7)	38 (18.5)	²0.480
≥ 60 years, n (%)	181 (82.3)	14 (93.3)	167 (81.5)	
Cancer stage, n (%)				
Stage III	29 (13.2)	4 (26.7)	25 (12.2)	^2^0.118
Stage IV	191 (86.8)	11 (73.3)	180 (87.8)	
Sex [n (%)]				
Male	186 (84.5)	11 (73.3)	175 (85.4)	³0.213
Female	34 (15.5)	4 (26.7)	30 (14.6)	
BMI				
Median (IQR)	22.3 (20.2–23.7)	23.2 (20.2–24.9)	22.3 (20.2–23.7)	^1^0.227
Underweight (BMI<18.5 kg/m²), n (%)	24 (10.9)	1 (6.7)	23 (11.2)	^4^0.295
Normal (18.5–23.9 kg/m²), n (%)	146 (66.4)	9 (60.0)	137 (66.8)	
Overweight (≥24 kg/m²), n (%)	50 (22.7)	5 (33.3)	45 (22.0)	
Patient history				
History of allergy, n (%)				
Yes	17 (7.7)	1 (6.7)	16 (7.8)	^2^1.000
No	203 (92.3)	14 (93.3)	189 (92.2)	
Medical history, n (%)				
None	56 (25.5)	4 (26.7)	52 (25.4)	^3^0.942
Pulmonary disease	25 (11.4)	1 (6.7)	24 (11.7)	
Cardiovascular disease	101 (45.9)	7 (46.7)	94 (45.9)	
Pulmonary and cardiovascular disease	38 (17.3)	3 (20.0)	35 (17.1)	
Autoantibodies (n = 209), n (%)				
Negative	119 (56.9)	5 (33.3)	114 (58.8)	³0.055
Positive	90 (43.1)	10 (66.7)	80 (41.2)	
Treatment-related				
Number of drug administrations, n (%)				
1 cycle	50 (22.7)	0 (0.0)	50 (24.4)	^4^0.043 ^5^0.085
2 cycles	99 (45.0)	8 (53.3)	91 (44.4)	
≥ 3 cycles	71 (32.3)	7 (46.7)	64 (31.2)	
Premedication, n (%)				
Two-drug combination	82 (37.3)	5 (33.3)	77 (37.6)	^4^0.717
Three-drug combination	86 (39.1)	6 (40.0)	80 (39.0)	
Four-drug combination	52 (23.6)	4 (26.7)	48 (23.4)	

¹Non-parametric test, ²Fisher’s exact test, ³Pearson’s chi-square test, ^4^Chi-square test for linear trend, ^5^Overall chi-square test.

### 3.2 Clinical characteristics of acute HSRs

**Figure 1:**
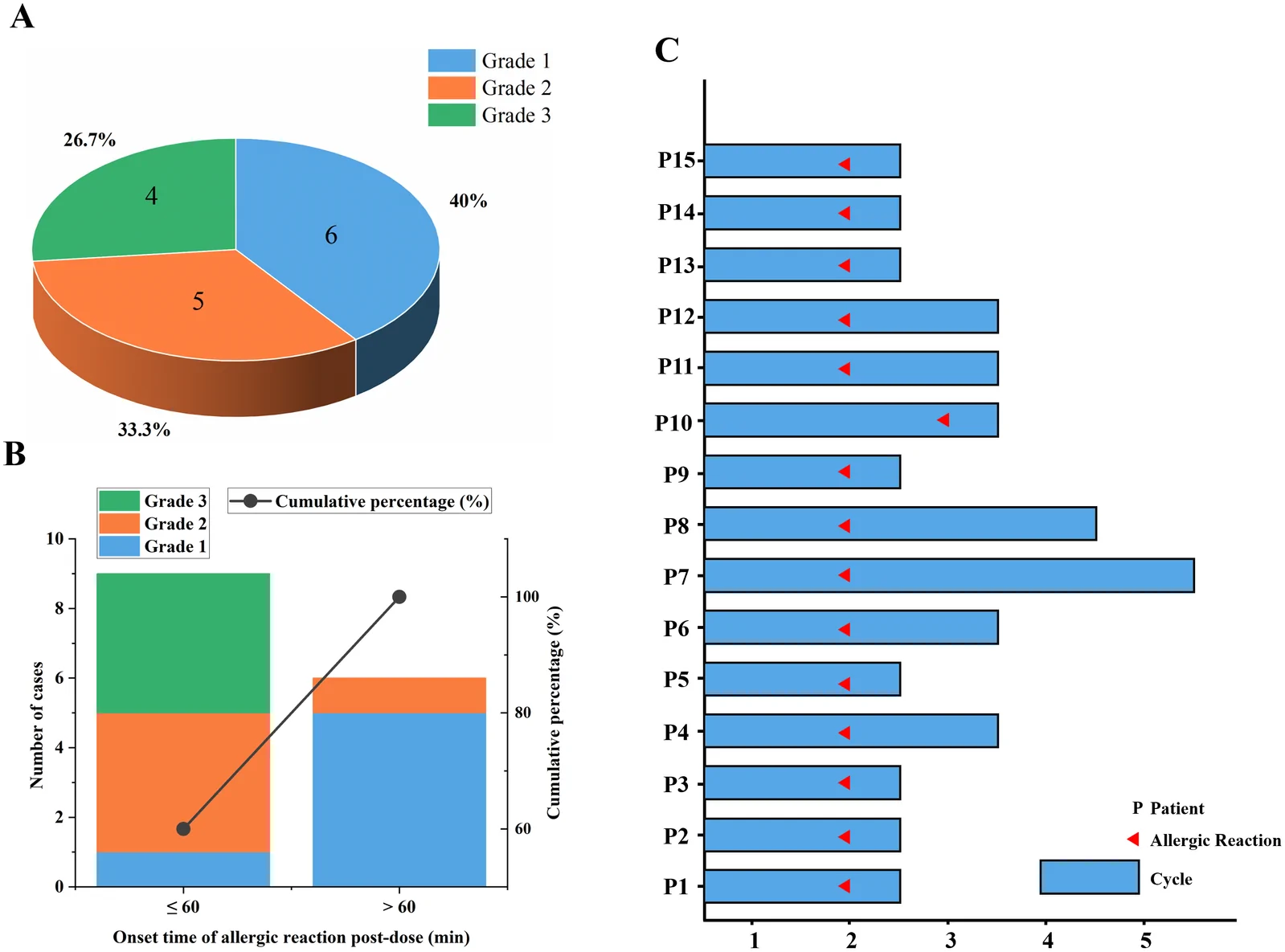
Characteristics of allergic reactions in patients receiving treatments. **(A)** Severity distribution of allergic reactions (Grade 1-3). The number of cases and corresponding percentages are shown for each grade. **(B)** Case counts and cumulative percentages of reactions by onset time(≤60 min vs. >60 min). **(C)** Distribution of allergic reactions across treatment cycles. Each red triangle represents an individual patient who experienced an allergic reaction during the indicated cycle.

The severity grades and clinical manifestations of the 15 patients with HSRs are detailed in **Table 2**. Grade 1 (mild) reactions occurred in 6 patients (40.0%), primarily as rash, pruritus, palpitations, and facial flushing. Grade 2 (moderate) reactions occurred in 5 patients (33.3%), involving ≥2 organ systems, including dizziness accompanied by nausea, rash, and conjunctival congestion, without hypotension or hypoxia. Grade 3 (severe) reactions occurred in 4 patients (26.7%) and were characterized by hypotension, dyspnea, or syncope, indicating potentially life-threatening risks (**Figure 1A**). Regarding the temporal distribution of hypersensitivity onset, 9 patients (60.0%) developed reactions within 60 min, and 6 (40.0%) developed reactions after 60 min (**Figure 1B**). Of the nine reactions that occurred within 60 min, eight (8/9, 88.9%) were grade ≥2. The median onset time category was >60 min, and the distribution across time intervals did not differ significantly (goodness-of-fit χ² = 2.333, *P* = 0.506). **Figure 1C** illustrates the specific treatment cycles in which HSRs occurred among the 15 patients. Fourteen reactions (93.3%, 14/15) occurred during the second treatment cycle and one (6.7%, 1/15) during the third cycle; no further HSR occurred during subsequent pm-Pac cycles among the six patients who continued treatment.

**Table 2:** Severity grading of hypersensitivity reactions.

Grade	Criteria	Number of cases	Proportion (%)
Grade 1	Systemic intervention not indicated: cutaneous or mild mucosal symptoms only (rash, pruritus, palpitations, facial flushing)	6	40.0%
Grade 2	Oral intervention indicated: involvement of ≥2 organ systems (skin and respiratory/cardiovascular/digestive/ocular, etc.), without hypotension or hypoxia	5	33.3%
Grade 3	Bronchospasm; hospitalization indicated for clinical sequelae; IV intervention indicated: hypotension, dyspnea, or syncope, indicating a life-threatening risk	4	26.7%

### 3.3 Risk factors for acute hypersensitivity reactions

#### 3.3.1 Chi-square tests

As shown in **Table 1**, a significant linear trend was observed between the number of pm-Pac administrations and the occurrence of HSRs (linear-by-linear association χ² = 4.082, *P* = 0.043). Regarding cancer stage, in the HSR group, 4 patients (4/15, 26.7%) were classified as stage III (intermediate-to-advanced) and 11 patients (11/15, 73.3%) as stage IV (advanced). In the non-HSR group, 25 patients (25/205, 12.2%) were classified as stage III and 180 patients (180/205, 87.8%) as stage IV. The difference in proportions between the groups was not statistically significant (Fisher’s exact test, *P* = 0.118), suggesting that cancer stage was not a significant predictor of acute HSRs to pm-Pac in this exploratory analysis.

The remaining variables were not significantly associated with HSR occurrence in our cohort, including sex (continuity-corrected chi-square test, *P* = 0.213), age dichotomized at 60 years (Fisher’s exact test, *P* = 0.480), BMI category (Pearson’s chi-square test, *P* = 0.295), history of allergies (Fisher’s exact test, *P* = 1.000), past medical history (Pearson’s chi-square test, *P* = 0.942), and premedication regimen (two-drug, three-drug, or four-drug combination; Pearson’s chi-square test, *P* = 0.717). The autoantibody positivity rate was 60.0% in the HSR group versus 41.2% in the non-HSR group (*P* = 0.055). Although this difference did not reach the conventional *α* = 0.05 threshold, the 18.8% absolute difference and the biological plausibility of immune dysregulation as a predisposing factor warrants further investigation in larger cohorts.

#### 3.3.2 Univariate logistic regression

Univariate logistic regression was used to explore associations between clinical variables and HSR status (**Table 3**). None of the evaluated clinical variables were significantly associated with HSR status, consistent with the baseline comparisons in **Table 1**. The association between the number of pm-Pac cycles and HSR status could not be estimated using logistic regression because the model failed to converge; no HSR events occurred in patients who received only one cycle of pm-Pac, so a reliable odds ratio could not be obtained. Nevertheless, the chi-square test for linear trend suggested an increasing trend of HSR occurrence with a greater number of administrations (*P* = 0.043).

**Table 3:** Results of the univariate logistic regression analysis.

Variable	β (regression coefficient)	Standard error	Wald χ²	*P* value	OR (95% CI)
Age	0.022	0.036	0.371	0.542	1.02 (0.95–1.10)
Sex	-0.752	0.616	1.488	0.222	0.47 (0.14–1.58)
Cancer stage	0.962	0.622	2.397	0.122	2.62 (0.77–8.85)
History of allergy	-0.170	1.067	0.025	0.874	0.84 (0.10–6.83)
Medical history	-0.049	0.257	0.036	0.848	0.95 (0.58–1.58)
Autoantibodies	-0.760	0.547	1.930	0.165	0.47 (0.16–1.37)

The dependent variable was “hypersensitivity status” (yes/no).

The independent variables were binary categorical. Given the limited number of events (15 cases in the allergy group), the confidence intervals may be unstable.

In this exploratory analysis, no significant predictors of HSRs to pm-Pac were identified among age, sex, cancer stage, history of allergy, comorbidities, or premedication regimens. However, given the limited number of events (n = 15), these negative findings should be interpreted with caution, as associations may be detectable in larger cohorts.

### 3.4 Dynamic changes in laboratory parameters

To evaluate trends in laboratory parameters across repeated measurements between the HSR and non-HSR groups, a linear mixed-effects model was fitted with group, time point (measurement number), and their interaction term were included as fixed effects and individual patient as a random effect. Results are presented in **Table 4**. BMI changed significantly over treatment cycles (F = 2.263, *P* = 0.038), but no significant group difference or group-by-time interaction was observed (*P* = 0.797 and *P* = 0.498, respectively). For C-reactive protein (CRP), the main effect of group was marginally significant (F = 3.381, *P* = 0.067). In contrast, the main effect of time point (*P* = 0.951) and the interaction effect (*P* = 0.742) were not significant. For albumin, eosinophils, basophils, white blood cell count, alanine aminotransferase (ALT), and aspartate aminotransferase (AST), neither the main effects (group or time point) nor the interaction effects reached statistical significance (*P* > 0.05). For creatinine, the main effect of time point was marginally significant (F = 2.092, *P* = 0.054), whereas the main effect of group (*P* = 0.177) and the interaction effect (*P* = 0.708) were not significant.

**Table 4:** Fixed-effects tests from linear mixed-effects models for each laboratory parameter.

Parameter	Fixed effect	Numerator df	Denominator df	F value	*P value*
BMI	Group	1	278.77	0.066	0.797
	Time point	6	287.58	2.263	0.038
	Group × Time point	4	288.22	0.845	0.498
CRP	Group	1	408.25	3.381	0.067
	Time point	6	299.77	0.268	0.951
	Group × Time point	4	303.29	0.492	0.742
Albumin	Group	1	418.10	0.357	0.550
	Time point	6	318.52	0.793	0.576
	Group × Time point	4	321.66	0.671	0.655
Eosinophils	Group	1	350.03	0.207	0.649
	Time point	6	470.07	0.150	0.989
	Group × Time point	4	461.62	0.216	0.929
Basophils	Group	1	408.91	0.222	0.638
	Time point	6	437.82	0.200	0.977
	Group × Time point	4	435.40	0.107	0.980
White blood cell count	Group	1	402.15	0.058	0.810
	Time point	6	310.50	0.705	0.646
	Group × Time point	4	313.95	0.444	0.777
Creatinine	Group	1	320.49	1.832	0.177
	Time point	6	288.32	2.092	0.054
	Group × Time point	4	289.60	0.537	0.708
ALT	Group	1	357.50	0.193	0.661
	Time point	6	404.11	0.399	0.880
	Group × Time point	4	400.17	0.439	0.781
AST	Group	1	414.93	0.133	0.716
	Time point	6	378.26	0.288	0.943
	Group × Time point	4	379.81	0.515	0.725

### 3.5 Management and outcomes of hypersensitivity reactions

The management measures and outcomes of the 15 patients with HSRs are detailed in **Table 5**. Among the 6 patients with Grade 1 reactions, none showed improvement with any treatment regimen (0%, 0/6). All 5 patients with Grade 2 reactions received systemic corticosteroids either alone or in combination with antihistamines and calcium gluconate, and all 5 improved (100%, 5/5). Among the 4 patients with Grade 3 reactions, 1 was excluded because treatment was discontinued; of the remaining 3, 2 improved after receiving combination therapy (66.7%, 2/3).

**Table 5:** Management and outcomes of hypersensitivity reactions.

Severity grade	Intervention after acute HSR	Recurrence after continued therapy		Outcome (%)	Notes	*P value*
		Yes (%)	No (%)			
I (n = 6, 40%)	No medication	1	0	0		^1^0.001
	Antihistamine alone	1	0			
	Antihistamine + calcium gluconate	1	0			
	Systemic corticosteroid alone	1	0			
	Systemic corticosteroid + antihistamine	1	0			
	Systemic corticosteroid + antihistamine + calcium gluconate	1	0			
II (n = 5, 33.3%)	Systemic corticosteroid alone	0	1	100		
	Systemic corticosteroid + antihistamine + calcium gluconate	0	4			
III (n = 4, 26.7%)	Systemic corticosteroid + antihistamine + calcium gluconate	1	2	66.7	One patient did not continue treatment	

1 Fisher’s exact test.

Fisher’s exact test was used to analyze the association between HSR severity grade (1, 2, 3) and treatment outcome (improved vs. not improved). As shown in **Table 5**, a significant association was observed between grade and outcome (Fisher’s exact test, *P* = 0.001), suggesting that severity grade may be associated with treatment response. Patients with Grade 1 reactions were less likely to improve, whereas all patients with Grade 2 reactions achieved favorable outcomes. Among the 8 patients who received the combination regimen comprising systemic corticosteroids, antihistamines, and calcium gluconate, 1 was excluded from the efficacy assessment because treatment was discontinued after the HSR. Of the remaining 7 patients, 6 achieved improvements (85.7%, 6/7).

## 4 Discussion

Although pm-Pac eliminates Cremophor EL and thus reduces the risk of non-immune-mediated HSRs in theory, the characteristics of immune-mediated HSRs to pm-Pac remain poorly described. To our knowledge, this is the first real-world retrospective study to systematically characterize the clinical features of acute HSRs to pm-Pac alongside dynamic laboratory changes. In this study, the overall incidence of acute HSRs was 6.8%, which likely reflects the heterogeneity of real-world clinical practice, including differences in patient comorbidities, combination treatment regimens, pretreatment strategies, and monitoring criteria. Mild-to-moderate reactions accounted for 73.3% of cases, with rash and pruritus as the predominant manifestations. The number of treatment cycles showed a significant linear trend with HSR rate (*P =* 0.043), whereas the overall chi-square test was borderline significant (*P =* 0.085). None of the traditional risk factors examined (age, sex, cancer subtype, or history of allergy) were significantly associated with HSR occurrence. Additionally, routine laboratory parameters did not differ between the HSR and non-HSR groups, suggesting that these parameters may not be suitable for HSR monitoring.

The incidence of acute HSRs to pm-Pac (6.8%) was lower than the 10.6% (152/1,430) reported by Taryn Boucher et al. for conventional paclitaxel (18) and the 10%–25% range cited in the literature (19), yet higher than the 1.3% incidence reported in phase III clinical trials (15). These differences may be attributable to the limited sample size of the present study and the inherent heterogeneity of real-world clinical practice. The pathophysiology of paclitaxel-induced HSRs remains incompletely understood. The current prevailing hypothesis involves a type I hypersensitivity reaction, in which the drug triggers IgE-mediated degranulation of mast cells and basophils, releasing histamine, leukotrienes, and other inflammatory mediators, thereby causing vasodilation, bronchospasm, and capillary leakage (20). In addition, the solubilizer Cremophor EL used in conventional paclitaxel formulations can directly activate the complement system, eliciting non-immune-mediated HSRs (21). Micellar formulations may reduce nonspecific mast cell degranulation induced by Cremophor EL used in conventional formulations (22). Furthermore, routine premedication with glucocorticoids and antihistamines in our center may have contributed to reducing HSR risk (23). Population heterogeneity might also influence susceptibility to HSRs (24). In terms of clinical presentation, cutaneous symptoms (rash and pruritus) were the most common (80%), consistent with the literature (25). Severe HSRs (grade 3) accounted for 26.7% of cases and occurred exclusively within the first hour. One patient (1/15, 6.7%) experienced a life-threatening event (blood pressure 66/44 mmHg). These findings indicate that although pm-Pac has a generally favorable safety profile, clinicians should remain vigilant for severe HSRs.

Regarding the cumulative effect of treatment cycles, a significant linear trend was observed (*P* = 0.043), whereas the overall chi-square test was borderline (*P* = 0.085). This discrepancy is not uncommon in analyses of ordinal categorical variables, as the linear-by-linear association test is more sensitive to monotonic trends (26). Clinically, this finding suggests a mild increase in HSR risk with an increasing number of pm-Pac administrations. However, the conventional significance threshold was not met in the overall chi-square test. We interpret this as a “dose-response trend” or a “cumulative effect signal”. Previous studies have indicated that the risk of chemotherapy-induced HSRs is closely related to cumulative drug exposure (27). Notably, all 15 HSR events occurred exclusively during the second or third treatment cycle, and no recurrence was observed in any subsequent cycle. Although this observation is consistent with the well-documented phenomenon of spontaneous desensitization observed with taxane chemotherapy (20, 28), whether this represents true tolerance induction or merely reflects the limited number of patients remains unclear. Unlike platinum-based chemotherapy, which typically causes HSRs after multiple cycles, taxane HSRs are predominantly non-IgE mediated and may resolve spontaneously with continued exposure (29). Our findings suggest that a similar phenomenon may occur with pm-Pac, but this observation requires validation in larger cohorts.

The negative findings regarding laboratory parameters are clinically meaningful. Linear mixed-effects models analysis showed that none of the eight laboratory indicators (white blood cell count, ALT, AST, albumin, eosinophil count, basophil count, creatinine, and CRP) differed significantly or showed distinct trends between the HSR and non-HSR groups. Although the group effect for CRP (*P* = 0.067) and the time effect for creatinine (*P* = 0.054) were borderline, these findings may reflect random fluctuations due to the limited sample size and exploratory nature of the analyses. These results suggest that routine hematological, hepatic, renal, and inflammatory parameters may not be useful for predicting or monitoring acute HSRs to pm-Pac (30, 31). Several factors may explain this observation: (1) premedication with glucocorticoids and antihistamines rapidly suppresses the release of inflammatory mediators, thereby attenuating allergy-related laboratory changes; (2) the retrospective design meant that laboratory testing times were not strictly aligned with the onset of HSRs, so true dynamic signals may have been masked by noise (32); (3) the small sample size of the allergic group (n = 15) provided insufficient statistical power to detect small differences. Therefore, clinical monitoring should rely primarily on symptom surveillance, with laboratory tests serving only as adjunctive information.

In terms of risk factor analysis, unlike Thangwonglers et al. (25), who reported age < 54.5 years as an independent risk factor, we found no significant association between traditional risk factors (age, sex, cancer subtype, history of allergy, comorbidities, premedication regimen) and HSR status. This discrepancy may stem from differences in sample size (only 15 HSR events in our study *vs.* 74 in Thangwonglers et al. (25)), study populations, drug formulation (pm-Pac vs. conventional paclitaxel), and premedication regimens. Thus, in our exploratory cohort, these variables were not identified as useful screening indicators for pm-Pac HSR. Emerging evidence indicates that preexisting autoantibodies may be associated with increased risk of immune-mediated adverse reactions to various oncology drugs, including immune checkpoint inhibitors and antibody-drug conjugates (33, 34). Autoantibodies may reflect underlying immune dysregulation that primes the innate immune system for exaggerated responses to chemotherapy agents. In our cohort, although the autoantibody positivity rate was numerically higher in the HSR group than in the non-HSR group, the difference did not reach statistical significance (*P* = 0.055) likely due to the limited number of observed events. Nevertheless, whether this numerical difference reflects a true biological association or a chance finding requires further investigation in larger cohorts.

In this study, severity grading of acute HSRs to pm-Pac was significantly associated with treatment outcome (*P = *0.001). With glucocorticoid-based regimens, grade 2 patients achieved 100% improvement, those with grade 3 reactions achieved 66.7%, whereas grade 1 patients showed 0% improvement despite various interventions. This apparent paradoxical finding may be explained by intermittent mild symptom recurrence in grade 1 patients, who were classified as “no improvement” (35). In contrast, more pronounced grade 2/3 reactions prompted more aggressive and consistent management. However, confounding by indication cannot be ruled out, as more severe reactions received more intensive treatment, so the observed improvement cannot be attributed solely to the glucocorticoid-antihistamine-calcium gluconate regimen. These findings are exploratory and hypothesis-generating, and confirmatory prospective controlled trials with adequate statistical power are needed.

Several limitations of this study should be acknowledged. First, the retrospective design may introduce selection and information bias; records of HSR severity and symptoms relied on medical documentation and lacked standardized assessment. Second, with only 15 HSR events, the statistical power was substantially limited, which precluded multivariate logistic regression analysis and reduced the reliability of univariable findings. Third, skin testing or drug provocation testing was not performed, so the culprit agent could not be definitively confirmed. Fourth, autoantibody testing was performed only in a subset of patients (n = 209), and we did not measure specific autoantibody titers or characterize the autoantigen specificity. This limits our ability to fully validate the observed trend and understand the underlying mechanisms. Future prospective studies should include comprehensive autoantibody profiling at baseline and serial measurements during treatment to clarify their predictive value and temporal relationship with HSR development. Future prospective, multicenter, larger-sample studies are also needed to validate our overall findings. Finally, while the HSRs observed were consistent with taxane-induced reactions (occurring predominantly during the second cycle (36), whereas platinum-associated HSRs typically develop after multiple cycles, often after more than six cycles (37), and cases attributed to other drugs were excluded by clinical assessment, the potential contribution of concomitant medications cannot be completely excluded in this real-world combination chemotherapy setting. This is an inherent limitation of retrospective real-world studies.

Based on these limited exploratory findings, the following considerations may be useful for clinical practice when interpreted with caution. First, although the incidence of HSRs to pm-Pac is low, close monitoring of symptoms within the first hour after infusion initiation is prudent, particularly from the second cycle onward, given the mild upward trend in HSR risk with increasing cycle number. Notably, in this cohort, patients who experienced mild-to-moderate HSRs during the second cycle were able to continue treatment with standard premedication and close monitoring, suggesting that continuing therapy may be a reasonable option for similar patients, although this observation requires confirmation in larger studies. This approach may avoid unnecessary discontinuation of an effective chemotherapy regimen. Second, routine laboratory tests are not suitable for predicting or monitoring HSRs; clinical judgment should rely on symptoms and signs. Regarding autoantibody positivity, the observed numerical difference did not reach statistical significance (*P =* 0.055), and the small number of events (n = 15) precludes any meaningful conclusion; this finding is presented as a purely exploratory observation rather than a suggested clinical tool. Third, patients requiring multiple administrations should be informed of the mildly increased HSR risk with increasing cycles, and standard premedication should be administered before each infusion. Fourth, once an HSR occurs, the infusion should be stopped immediately, and management should be guided by HSR grade using antihistamines, glucocorticoids, or epinephrine as appropriate. In this study, all patients with HSRs recovered completely after standard management with no sequelae.

## 5 Conclusions

In this cohort study, HSRs to pm-Pac were predominantly mild-to-moderate cutaneous manifestations, with an overall incidence of 6.8%. The autoantibody positivity rate was higher in the HSR group than in the non-HSR group; however, possibly due to the limited sample size, this difference did not reach statistical significance (*P* = 0.055). Given the small number of events (n = 15), this finding should be considered exploratory, and any potential clinical utility would require prospective validation. In the present study, all patients received premedication regimens comprising glucocorticoids and antihistamines, with either calcium supplements or H2-receptor antagonists. The relatively low incidence of HSRs observed in this cohort supports the continued use of prophylactic premedication before pm-Pac infusion. During follow-up of up to seven treatment cycles, most HSRs occurred in the second cycle, with one in the third, suggesting that monitoring may need to be extended beyond the second cycle. Collectively, these findings provide clinical guidance for managing pm-Pac–induced HSRs in patients with malignancies.

Several directions for future research emerge from this exploratory study. First, prospective cohorts with larger sample sizes are needed to validate the observed trend between baseline autoantibody positivity and pm-Pac HSR risk and to identify the specific autoantibody subtypes most strongly associated with HSR occurrence. Second, mechanistic studies are warranted to elucidate whether the absence of recurrent HSRs in subsequent cycles reflects true spontaneous desensitization (e.g., mast cell mediator depletion or induction of regulatory immune responses) or is attributable to the limited sample size. Serial measurements of mast cell mediators, such as tryptase and histamine, before and after repeated pm-Pac exposure could help clarify this mechanism. Third, randomized controlled trials comparing different premedication regimens are needed to establish evidence-based protocols for the prevention and management of HSRs. Fourth, the potential utility of skin testing in predicting pm-Pac HSRs or guiding desensitization strategies should be explored.

## Data Availability

The original contributions presented in the study are included in the article/supplementary material. Further inquiries can be directed to the corresponding authors.
